# New amphiphilic glycopolymers by click functionalization of random copolymers – application to the colloidal stabilisation of polymer nanoparticles and their interaction with concanavalin A lectin

**DOI:** 10.3762/bjoc.6.58

**Published:** 2010-06-01

**Authors:** Otman Otman, Paul Boullanger, Eric Drockenmuller, Thierry Hamaide

**Affiliations:** 1 Université de Lyon; Université Lyon 1; Ingénierie des Matériaux Polymères, (IMP UMR CNRS 5223). 15, boulevard Latarjet, Villeurbanne, F-69622, France; 2 Université de Lyon; Université Lyon 1; Institut de Chimie et Biochimie Moléculaires et Supramoléculaires (ICBMS), Chimie Organique 2 – Glycochimie, UMR CNRS 5246, CPE-Lyon. 43, boulevard du 11 Novembre 1918, Villeurbanne, F-69622, France

**Keywords:** click chemistry, con A lectin, dynamic light scattering, glycopolymer, polymer nanoparticles

## Abstract

Glycopolymers with mannose units were readily prepared by click chemistry of an azido mannopyranoside derivative and a poly(propargyl acrylate-co-*N*-vinyl pyrrolidone). These glycopolymers were used as polymer surfactants, in order to obtain glycosylated polycaprolactone nanoparticles. Optimum stabilization for long time storage was achieved by using a mixture of glycopolymers and the non-ionic triblock copolymer Pluronic^®^ F-68. The mannose moieties are accessible at the surface of nanoparticles and available for molecular recognition by concanavalin A lectin. Interaction of mannose units with the lectin were evaluated by measuring the changes in nanoparticles size by dynamic light scattering in dilute media.

## Introduction

Over the last decades, research efforts in pharmaceutical, food and cosmetics technologies have been directed not only towards the syntheses of new bioactive entities or medicines, but also towards new formulations that can enhance the activity of drugs, as well as the elaboration of new drug delivery systems. The main objectives are the transport of active hydrophilic or lipophilic substances, while minimizing drug degradation, increasing drug availability and localization in the required organ. At the same time, the development of “all in one” formulations encourages manufacturers to introduce more and more active components in the finished products, so that new systems are nowadays widely investigated in order to get synergies in one product, to propose innovative properties and to combine immediate and delayed effects, through the selection of an efficient drug and the design of a suitable dosage form. Encapsulation of hydrophobic substances in aqueous dispersed media can be performed by a number of methods. Notably, synthetic polymer-based nanoparticles have received considerable attention because of their potential since they represent attractive alternatives to conventional pharmaceutical applications. In addition, the size, morphology and composition of the polymer particles can be tuned to optimize the drug release kinetics in order to reduce, e.g., toxicity and improve efficacy. These parameters are closely connected. For instance, the chemical composition of the polymer matrix may affect the particle morphology because of thermodynamic interactions between the hydrophobic drug and the polymer.

Whatever the drug delivery system, surfactants, and in particular polymer non-ionic surfactants, are required to assure the colloidal stabilization of the polymer nanoparticles in aqueous medium. The properties of micelles (cmc, size and dynamics) depend on the chemical structures of amphiphilic copolymers. Macromolecular non-ionic surfactants appear to be the best suited from both the stability and the biological points of view. In such block copolymers, the hydrophobic part is enhanced in comparison to molecular surfactants, which allows a better adsorption (by reducing the exchange dynamics) and increases the long term stability. This stronger adsorption also reduces the residual concentration of free surfactant in the aqueous phase. The hydrophilic part is most often constructed from PEG chains. In addition to the steric stabilization, this PEG coating reduces the detection of particles or liposomes by the immune system and consequently, the reticular endothelial uptake of nanoparticles, thus increasing their circulation in the body. The polyether triblock copolymer PEO-b-PPO-b-PEO Pluronic^®^ F-68 (PF-68) is approved by the US Food and Drug Administration and is thus widely used in pharmaceutical formulations.

Glycopolymers can advantageously replace these block copolymers. In addition to the hydrophilicity conferred by the carbohydrate moiety, specific targeting may be result from coating nanoparticles with oligo- or polysaccharide chains since the carbohydrate moieties play an essential role in molecular recognition processes. Although the carbohydrate ligands occur naturally as glycoconjugates or as high molecular weight polymers, the actual “recognized” fractions are most often small oligosaccharides (3 to 10 carbohydrate units). Efficient binding has also been reported for monosaccharide ligands; for instance, galactose residues are targetable moieties for hepatocytes [1] whilst mannose units can be used for nerve cells targeting [2]. Besides naturally occurring polysaccharides, macromolecular engineering allows quite interesting possibilities for modelling of synthetic glycopolymers [3].

The hydrophobic component can be introduced by any convenient polymer moiety. Some of these are obtained using methodologies based on living polymerizations in order to achieve controlled molecular weights and narrow polydispersity indexes. Depending on the chemistry, carbohydrate moieties can be grafted either at the polymer chain end or as pendant groups on the polymer backbone. Thus, polycaprolactones (PCL) functionalized with galactopyranose end-groups have been synthesized by ring opening polymerization and used for the stabilization of PCL nanoparticles [4]. Glucose or cellobiose moieties have been grafted at both ends of short PDMS chains and used as polymer surfactants for mini-emulsion polymerization [5–6]. Amphiphilic block copolymers with pendant glucosamine units have been obtained by living cationic polymerization and their interaction with wheat germ agglutinin lectin investigated [7]. More recently, the synthesis of neoglycopolymers by living radical polymerization and click chemistry has been reported [8–9].

Besides homopolymers or block copolymers, statistical copolymers obtained from conventional radical polymerization deserve special attention because of their ease of synthesis. Well controlled MW as well as narrow PI are not always pre-requisites to achieve interesting colloidal stabilization properties. Carbohydrate residues such as galactosyl moieties have also been incorporated as side groups through the ring opening of maleic anhydride based copolymers [10–11]. We recently reported the synthesis and characterization of amphiphilic copolymers bearing carbohydrate and oligocaprolactone side chains, obtained via copolymerization of a PCL macromonomer and maleic anhydride, and further functionalization by ring opening of the anhydride moiety with amino mannopyranoside. These copolymers were then used as polymer surfactants for the stabilization of PCL nanoparticles coated with carbohydrate on their surface [12].

This paper reports the first results on another class of glycopolymers obtained from the functionalization of poly(propargyl acrylate-co-*N*-vinyl pyrrolidone) by click chemistry with ω-mannopyranoside. These copolymers were used as polymer surfactants for the colloidal stabilization of polycaprolactone (PCL) nanoparticles. Preliminary studies towards the recognition of these glyco-nanoparticles by specific concanavalin A (con A) lectin have been carried out, which provided clear evidence for the presence of carbohydrate units on the surface of the nanoparticles

## Results and Discussion

### Synthesis and characterization of the polymer surfactants

Besides well-known functionalization of anhydride-based copolymers, the grafting of carbohydrate moieties on a polymer backbone by Huisgen [2 + 3] cycloaddition (CuI-catalyzed 1,3-dipolar cycloaddition of azide and alkynes, CuAAC) constitutes another interesting approach. The versatile nature of this reaction has led to a tremendous amount of work, mainly due to the quantitative yields and the possibility of carrying out the synthesis in either organic solvents or water. Moreover, since various other functional groups can be tolerated, one-pot tandem approaches can be considered [13]. Similarly, in the carbohydrate field, this unique feature makes it possible to conduct syntheses by simplified pathways without protection–deprotection steps. We chose to use poly(propargyl acrylate-co-*N*-vinyl pyrrolidone) as the starting copolymer. Poly(NVP) is known to be biocompatible and to promote adhesion. NVP-based maleic copolymers have been reported for BSA immobilization [14] as well as for the preparation of polymer nanoparticles [10,15].

The choice of the carbohydrate moieties to be grafted onto the copolymer and their related syntheses were based on the following criteria: 1 - the monosaccharide must be anchored onto the polymer backbone by the use of a hydrophilic spacer in order to allow more freedom inside the aqueous phase after adsorption onto the polymer particles; 2 - syntheses must be simple enough to allow scale-up.

**Figure 1:**
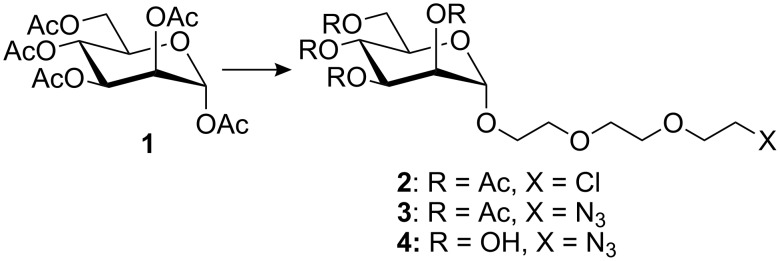
Preparation of the 8-azido-3,6-dioxaoctyl α-D-mannopyranoside.

Thus, we elected to use the simplest carbohydrates, i.e., those which are easy to prepare and easy to handle, e.g., the peracetylated monosaccharide **1** [16]. Although other derivatives, such as the 2,3,4,6-tetra-*O*-acetyl-1-*O*-trichloroacetimidoyl mannose might give rise to better glycosylation yields, the scale-up of these reactions was shown to be difficult. As a spacer, we chose triethylene glycol. The reaction of **1** with 8-azido-3,6-dioxaoctyloctan-1-ol (prepared in several steps from triethylene glycol) [17] was found to be unsatisfactory since the by-products formed in the reaction were difficult to separate from the desired compounds. The addition of triethylene glycol derivatives was also attempted by coupling triethylene glycol, or an activated triethylene glycol derivative bearing a tosylate or a chlorine atom at the ω-position. This latter approach proved to be better. The best result was obtained with commercially available triethylene glycol monochloride. Thus, peracetylated mannose **1** was reacted with triethyleneglycol monochloride, in the presence of boron trifluoride etherate to afford glycoside **2** [12] in 50–55% yield. After purification, the latter was converted to the azido derivative **3** in almost quantitative yield, by nucleophilic displacement of the chlorine atom by azide ion. Deprotection of the latter under Zemplén conditions [17] afforded the expected derivative **4** in quantitative yield (Figure 1).

The copolymer backbones, namely the poly(propargyl acrylate-co-*N*-vinyl pyrrolidone), were prepared by conventional radical copolymerization using propargyl acrylate and *N*-vinyl pyrrolidone as comonomers. The reactions were carried out in dry THF at 65 °C under an argon atmosphere with lauroyl peroxide as the initiator. The copolymers were obtained as a white powders by precipitation in diethyl ether which were dried under vacuum and characterized by IR, ^1^H and ^13^C NMR and SEC. Kinetics were also monitored by ^1^H NMR from the disappearance of the monomer signals. Yields were in the range of 60 to 90%. Although some radical transfer reactions leading to addition to the acetylene groups has been reported on reaching high yields [18], no gel formation was observed in our case during polymerization, so that protection of the propargyl monomer was not required. Molecular weights were around 10000 g/mol with *M*_w_/*M*_n_ ≈ 1.6–2.0. The molar fraction of PA units in the copolymer was in the range of 0.25 to 0.75.

**Figure 2:**
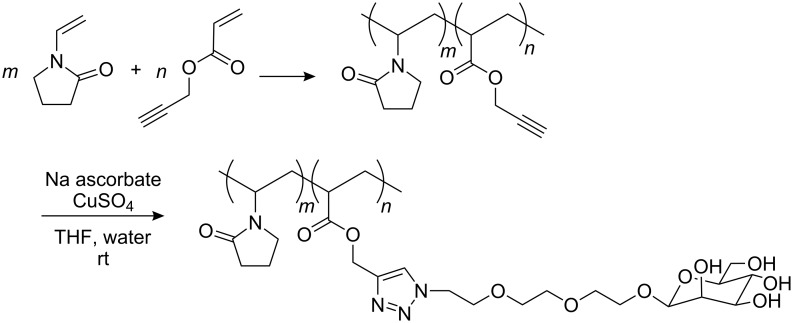
Preparation of poly(propargyl-co-*N*-vinyl pyrrolidone) and subsequent addition of the mannose derivative by click chemistry.

As previously outlined, coupling reactions with sugar moieties generally require protection and deprotection steps of the hydroxyl functions. Click chemistry can of course be performed with peracetylated mannose followed by deprotection, but this involves all the drawbacks inherent to polymer structures. Due to the high versatility of click chemistry in the presence of functional groups, it was then of interest to attempt the reaction with unprotected sugars. Moreover, this chemistry can be carried out not only in various organic solvents, but also in aqueous alcoholic media and water. In our case, the click reaction was performed with CuSO_4_ and sodium ascorbate in a THF/water mixture (Figure 2). This approach was preferred to the use of other catalysts because of the applications listed in the specific area of colloidal stabilization and the ease of removal of copper salts with ethyl xanthogenate or cation exchange resin. Moreover, sodium ascorbate is far easier to remove in contrast to DIPEA or bipyridine and additionally, it would not be of any great consequence if some residual sodium ascorbate remained in the medium. The resulting grafted copolymers were fully characterized by IR (disappearance of *ν*(N_3_) at 2121 cm^−1^ and *ν*(C≡C) at 2129 cm^−1^) and NMR: ^1^H (δ H_12_ at 8.06 ppm) and ^13^C (δ C_11_ and C_12_ respectively, at 143.32 and 125.82 ppm). DEPT experiments were used to ascertain the chemical shifts and to ensure the complete conversion of the azide functions. These copolymers are water soluble.

### Recognition of PCL nanoparticles by concanavalin A

Lectins are proteins of non-immunological origin, able to bind carbohydrate ligands, without any enzymatic or immunological function. They are multivalent and can bind several ligands simultaneously and participate in biological phenomena such as cell adhesion or cell–cell recognition. The specific binding of a lectin receptor with a carbohydrate ligand (usually called recognition) is obtained, provided that the ligands are orientated in a specific manner that can fit several lectin receptors and is usually referred to as the cluster effect [19–20]. The lectin–carbohydrate specificity strongly depends on the lectin. Several of these are very sensitive to the nature of the carbohydrate (e.g. Man vs Gal), whereas others are more sensitive to the orientation of the anomeric substituent (i.e. α vs β). Concanavalin A, used in the present work, belongs to the latter group and displays strong specificity towards α-D-mannopyranose. This lectin is dimeric and divalent at pH < 5.6 and tetrameric and tetravalent at pH > 5.6. Its specificity is directed to α-D-mannopyranosides and to a lesser extent to α-D-glucopyranosides and α-D-galactopyranosides, and shows no affinity at all towards β-D-monosaccharides [21].

Consequently, the addition of a tetravalent lectin to a suspension of nanoparticles covered with large amounts of its specific ligands should lead to agglutination of the particles, thus giving rise to flocculation or precipitation, whereas the suspension should remain unchanged on the addition of “naked” nanoparticles (without any carbohydrate on their surface) or nanoparticles covered with non specific carbohydrates. This increase in size can be observed either by the naked eye, turbidity or fluorescence measurements as previously reported in the literature [7,22]. Measurements by light scattering have only occasionally been reported in the literature [23].

Polycaprolactone nanoparticles were prepared according to the emulsification–diffusion procedure as proposed by Quintanar and Fessi [24–25] by using a mixture of PEO-b-PPO-b-PEO triblock copolymer (Pluronic^®^ F-68) and our functionalized copolymers as polymer surfactants. This technique is based on the rapid diffusion of the organic solvent from the internal into the external phase, which causes the precipitation of the polymer as colloidal nanoparticles. We previously used this procedure to obtain polycaprolactone nanoparticles with end-capped oligocaprolactones with galactopyranose moieties as polymer surfactants [4].

In this case, some previous studies have clearly shown that the glycopolymers employed alone are unable to assure colloidal stabilization of polymers in dispersed media. This behavior was observed when using other types of glycopolymers [6,12] where a second polymer surfactant was required to assure a stable colloidal stabilization upon long time storage. This was tentatively interpreted in terms of layer thickness of the polymer surfactant adsorbed onto the particles.

We used here Pluronics instead of poly(vinyl alcohol) (PVA), as proposed by Quintanar and Fessi, to get a higher solid content for the nanoparticles suspensions (the evaporation step rapidly leads to highly viscous suspensions because of the high molecular weight of PVA). Some other advantages of Pluronics for biomedical applications have already been detailed in the introduction. Nanoparticles stabilized by Pluronic^®^ F-68 and 20% (w/w) of the glycopolymers previously prepared were used in our investigations.

In a first set of experiments, increasing amounts (10 to 1000 µL) of a con A solution (8.7 mg/10 mL of PBS, pH 6.8) were added to nanoparticles stabilized by only Pluronic^®^ F-68. No changes in size were observed over a 2 h period irrespective of the con A concentration. Similarly, no significant change in size was recorded over 2 h on the addition of increasing amounts of con A to PCL particles stabilized by a mixture of Pluronic^®^ F-68 and the precursor poly(PA-co-NVP) (20% w/w).

The same results were observed when either crude suspensions of nanoparticles or their supernatants after centrifugation (2000 g; 1 h) were used. The same tendencies were also observed with the re-suspended pellets, but the results were not so relevant since more important variations in size were observed from one sample to another. Thus, the recognition experiments then conducted only with the supernatant fractions of the particle suspensions. All the observations that will be described were also true for crude suspensions as well as re-suspended pellets however, the supernatants are more homogenous and the more abundant fractions, and contain about 65 % (of the dry weight) of nanoparticles.

**Figure 3:**
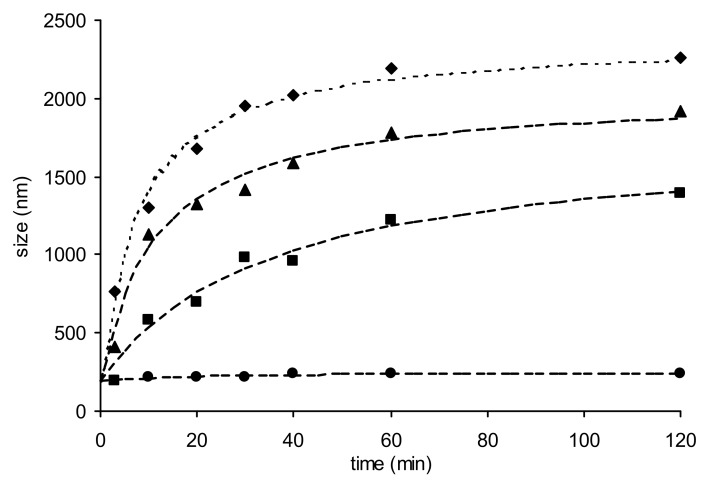
Size of the nanoparticles stabilized with Pluronic^®^ F-68/NVP-PA-Man (0.8/0.2), after addition of increasing amounts of con A. ● 10 μL; ■ 50 μL; ▲ 100 μL; ♦ 1000 μL. Measurements realized on supernatant of centrifuged nanoparticles.

This time increasing amounts of con A were added to the supernatant fraction of colloidal suspensions of nanoparticles stabilized by the mixture of PF-68 and NVP-PA-Man. A very important increase in size was observed, even with low amounts of lectin (50 µL). The kinetics of size increase was proportional to the amount of lectin added (Figure 3). Furthermore, flocculation occurred with the highest concentrations and the suspensions changed from clear colorless to milky. As can be seen from Figure 3, changes in size were drastic, giving rise to aggregates of almost 2 µm in diameter, i.e. ten times the size of isolated nanoparticles.

Particles covered with β-D-galactose were used as control experiments since concanavalin A has no affinity for this monosaccharide. Some of the experiments were repeated under the same conditions with nanoparticles covered with D-galactose, i.e. stabilized by a mixture of PF-68 and NVP-PA-Gal. No change in size was observed over 2 h (<30 nm) irrespective of the amount of added lectin. This result corroborates the previous assumption regarding the observations made with the first group of nanoparticles, i.e. the apparent non specific binding of the lectin can be attributed the formation of a covalent bond between the protein and the residual anhydride functions of the glycopolymer.

## Conclusion

Water soluble glycopolymers can easily be prepared by the CuI-catalyzed cycloaddition of an azido mannopyrannoside derivative and a poly(propargyl acrylate-co-*N*-vinyl pyrrolidone). These glycopolymers can be used as polymer surfactants, in conjunction with another non-ionic triblock copolymer in order to obtain glycosylated polycaprolactone nanoparticles stable over a long period of storage.

The carbohydrates covalently bound to the copolymers are accessible at the surface of the nanoparticles and are available for molecular recognition. The recognition of α-D-mannose at the surface of the nanoparticles by con A is specific, provided that no further reactive groups which could covalently bind the lectin are present on the polymer. In addition, light scattering appears to be the method of choice to detect carbohydrate recognition by a lectin at the surface of nanoparticles in dilute media. This approach can be easily extended to multifunctional polymer nanoparticles containing encapsulated drugs inside their core.

## Experimental

The syntheses of 8-chloro-3,6-dioxaoctyl 2,3,4,6-tetra-*O*-acetyl-α-D-mannopyranoside (**2**) and 8-azido-3,6-dioxaoctyl 2,3,4,6-tetra-*O*-acetyl-α-D-mannopyranoside (**3**) were reported in a previous paper [12].

### Materials

Polycaprolactone (MW 80000 g/mol), Pluronic^®^ F-68 and propargyl acrylate were purchased from Aldrich. *N*-Vinyl pyrrolidone and lauroyl peroxide were purchased from Fluka. Thin layer chromatography was performed on aluminium sheets coated with Silica gel 60 F_254_ (Merck). Compounds were visualized by spraying the TLC plates with dilute 15% aq. H_2_SO_4_, followed by charring at 150 °C for a few minutes. Column chromatography was performed on Silica-gel Geduran Si 60 (Merck). ^1^H and ^13^C NMR spectra were recorded with a Bruker DRX-300 spectrometer at 300 MHz and 75 MHz respectively, or with a Bruker DRX-500 spectrometer at 500 MHz and 125 MHz respectively, with TMS as internal standard. High resolution mass spectra were recorded with a ThermoFinnigan MAT95XL double focusing mass spectrometer equipped with an ESI III source.

### 8-Azido-3,6-dioxaoctyl α-D-mannopyranoside (**4**)

The protected derivative **3** (500 mg, 0.99 mmol) was dissolved in dry methanol (10 mL) and stirred for 1h at room temperature in the presence of a catalytic amount of sodium. The mixture was then neutralized with Amberlist IR 120 H^+^ resin, filtered and evaporated to afford compound **4** in quantitative yield as a clear oily material. [α]_D_ +38.7 (c 1.0, 25 °C, MeOH). *R*_f_ 0.49 (5:1, ethyl acetate/methanol). ^1^H NMR (D_2_O): δ (ppm) 4.79 (d, 1H, *J*_1,2_ 1.5 Hz, H-1), 3.86 (dd, 1H, *J*_2,3_ 3.6 Hz, *J*_3,4_ 9.2 Hz, H-3), 3.81 (t, 1H, H-4), 3.77 (dd, 1H, H-2), 3.63 (m, 12H, H-6, H-6′, (OC*H*_2_C*H*_2_)_2_C*H*_2_), 3.56 (m, 1H, H-5), 3.42 (t, 2H, C*H*_2_N_3_). ^13^C NMR (CD_3_OD): δ (ppm) 100.30 (C-1), 73.09 (C-5), 70.00, 69.92, 69.87 (O*C*H_2_*C*H_2_)_2_O*C*H_2_), 70.87 (C-2), 70.32 (C-3), 67.09 (C-4), 61.28 (C-6), 50.51 (*C*H_2_N_3_). HRMS (ESI) *m/z* Calcd for [C_12_H_25_O_8_N+Na]^+^ 360.13830; Found 360.13837.

### Poly(propargyl acrylate-co-*N*-vinyl pyrrolidone)

**Figure 4:**
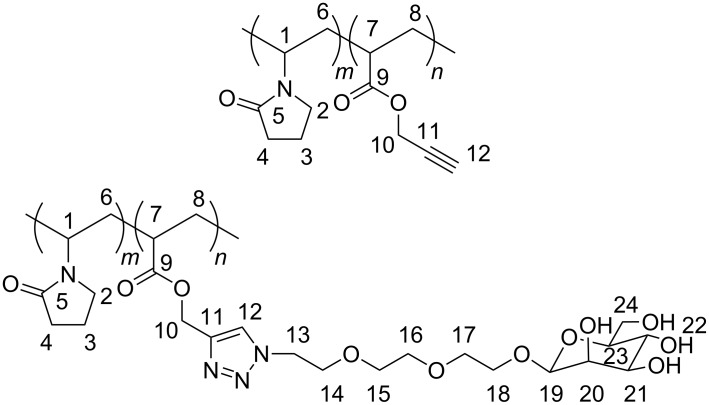
Hydrogen and carbon numbering for NMR assignment.

Copolymerizations were carried out in dry THF at 65 °C under an argon atmosphere with 3–5% of lauroyl peroxide as the initiator. For example, propargyl acrylate (0.309 g, 2.93 mmole) and *N*-vinyl pyrrolidone (0.095 g, 0.85 mmol) were dissolved in dry THF (20 mL) at 65 °C. The solution was degassed for 15 min with argon, before the addition of lauroyl peroxide (0.060 g, 0.15 mmol). After 8 h, the copolymer was recovered as a white powder by precipitation in diethyl ether (three times), dried under vacuum (85% yield) and characterized by IR (ν(C≡C) at 2129 cm^–1^), ^1^H and ^13^C NMR (Figure 4).

^1^H NMR (CDCl_3_, 300 MHz): δ (ppm) 5.75 (H-1); 4.5–4.95 (H-10); 3.6–4.2 (H-2); 3.25 (H-7); 2.55 (H-12); 2.35 (H-4); 1.3–2.1 (H-3, H-6, H-8). ^13^C NMR (CDCl_3_, 125 MHz): δ (ppm) 174.19–175.79 (C-5, C-9), 75.77 (C-11), 79.36 (C-12), 52.68 (C-10), 47.91 (C-1), 39.03 (C-78), 35.42 (C-2), 31.67 (C-4), 26.00–26.07 (C-6, C-8), 18.57 (C-3).

### Coupling of azido functionalized carbohydrates by CuAAC

The dry copolymer (100 mg, containing approximately 75 mg, 0.68 mmol of propargyl acrylate) and compound **4** (229 mg, 1.0 equiv vs propargyl acrylate) were dissolved in a 1:1 mixture of THF and water (4 mL) at room temperature. After the addition of CuSO_4_ (6 mg, 0.02 mmol) and sodium ascorbate (18 mg, 0.09 mmol), the mixture was stirred at room temperature for 12 h, the solution evaporated to dryness and dried under vacuum. The light green product thus obtained was dissolved in de-ionized water (5 mL) containing 50 mg of potassium ethyl xanthogenate to remove the copper salts. After filtration through celite and evaporation to dryness, the desired mannose-functionalized copolymer was obtained as a pale yellow solid in 73% yield.

^1^H NMR (CD_3_OD, 500 MHz): δ (ppm) 8.06 (H-12), 5.8 (H-1), 5.10 (H-10), 4.6–4.85 (4 OH, H-19), 4.56 (H-2), 3.50–4.10 (H-13/H-18, H-20/H-24), 3.10 (H-8), 2.35 (H-4), 1.20–2.10 (H-3, H-6, H-7). ^13^C NMR (CD_3_OD, 125 MHz): δ (ppm) 174.20–178.50 (C-5, C-9), 143.32 (C-11), 125.82 (C-12), 100.75 (C-19), 73.37 (C-20), 71.74 (C-21), 71.10 (C-23), 69.50 (C-15, C-16, C-17, C-18), 67.82 (C-22), 66.93 (C-14), 62.05 (C-24), 58.07 (C-10), 50.65 (C-13), 48.40 (C-1), 40.04 (C-7), 35.00 (C-2), 31.48 (C-4), 26.00–26.50 (C-6, C-8), 18.27 (C-3).

### Nanoparticle formation

Ethyl acetate and water were contacted for 2 h in order to obtain mutually saturated solutions. Polycaprolactone (80000 g/mol) (1.0 g) was dissolved in ethyl acetate (25 mL) under gentle stirring and heating for 2 h (ethyl acetate was added in order to adjust the volume to 25 mL). Pluronic^®^ F-68 and the glycopolymer, dissolved in water (50 mL), were then added to the preceding solution, the mixture stirred for 10 min and then emulsified by sonication (ultrasonic processor Vibra Cell VCX-750) for 2 min at 525 W. De-ionized water (175 mL) was then added while stirring the solution at 700–800 rpm. The nanoparticle suspension was then concentrated at atmospheric pressure to 30–35 mL by stirring and heating to 70–80 °C.

The sizes of nanoparticles were measured by dynamic light scattering (each measurement was repeated five times) at 25 °C with a Malvern Zetasizer 1000HSa series (wavelength 633.0 nm, RI (dispersant) 1.330, angle 90°); data treatments were made in intensity, with water viscosity 0.89 cP.

### Interaction of the nanoparticles containing D-mannose with con A

In a 20 mL flask, 8.7 mg of concanavalin A was dissolved without stirring in 10 mL of phosphate-buffered saline (PBS, pH = 6.8, 0.1 M) over 16 hours. The flask was then stored at 4 °C. The nanoparticle stock solution (200 mg of dry matter in 3.5 mL H_2_O) was diluted in PBS and various volumes of con A solution in the same buffer solution (10, 20, 50, 100, 200, 500, 1000 μL) were added; the final volume was adjusted to 5 mL by addition of PBS.
